# Experimentally Induced Bladder Permeability Evokes Bladder Afferent Hypersensitivity in the Absence of Inflammation

**DOI:** 10.3389/fnins.2020.590871

**Published:** 2020-10-23

**Authors:** Luke Grundy, Ashlee Caldwell, Amanda Lumsden, Ehsan Mohammadi, Gerhard Hannig, Beverley Greenwood Van-Meervald, Stuart M. Brierley

**Affiliations:** ^1^Visceral Pain Research Group, Flinders Health and Medical Research Institute (FHMRI), Flinders University, Bedford Park, SA, Australia; ^2^Hopwood Centre for Neurobiology, Lifelong Health Theme, South Australian Health and Medical Research Institute (SAHMRI), Adelaide, SA, Australia; ^3^Discipline of Medicine, University of Adelaide, North Terrace, Adelaide, SA, Australia; ^4^Oklahoma Center for Neuroscience, University of Oklahoma Health Science Center, Oklahoma City, OK, United States; ^5^V.A. Medical Center, Oklahoma City, OK, United States; ^6^Ironwood Pharmaceuticals, Boston, MA, United States

**Keywords:** bladder, afferent, interstitial cystitis, bladder pain syndrome, urothelium, permeability

## Abstract

Interstitial cystitis/bladder pain syndrome (IC/BPS) is a chronic urological condition characterised by urinary urgency, frequency and pelvic pain, that significantly impacts the quality of life for ∼5% of women. Bladder sensation is coordinated by primary afferent sensory neurons that innervate the bladder wall, translating bladder stretch into signals that travel to the brain via the spinal cord. Whilst the pathophysiology of IC/BPS remains unknown, an increase in the permeability of the bladder urothelium has been proposed as an initiating cause. Here we experimentally increased bladder permeability and tracked bladder afferent sensitivity for up to 28 days. We found that one day after increasing bladder epithelial permeability with *in vivo* bladder infusion of protamine sulfate, mechanosensitive bladder afferents exhibited significant hypersensitivity to bladder filling. This mechanical hypersensitivity was characterised by significantly increased peak afferent firing rates and a decrease in the activation threshold of individual afferents. Bladder afferent hypersensitivity occurred in the absence of inflammation and changes in bladder muscle compliance, indicating a direct sensitisation of peripheral afferent endings. Bladder afferent mechanosensitive responses to distension returned to control levels by day 7 post-protamine sulfate treatment and remained at control levels at 28-days post-treatment. Here we demonstrate, contrary to the prevailing hypothesis, that increased bladder permeability alone does not induce chronic bladder afferent sensitisation. Whilst experimentally induced changes in bladder permeability are able to induce transient bladder afferent hypersensitivity in the absence of inflammation, highly regulated homeostatic mechanisms exist to rapidly repair the urothelial barrier and normalise bladder afferent mechanosensitivity. Together, these data suggest that additional pathophysiology is required to induce chronic bladder dysfunction.

## Introduction

Interstitial cystitis/bladder pain syndrome (IC/BPS), is a common urological condition characterised by persistent or recurrent chronic pelvic pain that is often accompanied by urinary urgency and/or frequency (Grundy et al., 2018a; Akiyama et al., 2019). Whilst the pathophysiology of IC/BPS has not been objectively described and agreed upon, an increase in the permeability of the bladder urothelium has been proposed as a potential cause of the symptoms (Parsons, 2007; Keay et al., 2014; Hurst et al., 2015).

The bladder wall contains a dense network of afferent nerves found both in the detrusor smooth muscle, and in close proximity to the bladder lumen and urothelium (Kanda et al., 2016; Spencer et al., 2018). These afferents respond directly to stretch, exhibiting firing rates that closely encode intravesical pressure, and regulate the autonomic circuits responsible for bladder storage (Fowler et al., 2008; Grundy et al., 2019a,b). At greater bladder pressures, these afferent signals progress to the cerebellum, providing sensation and conscious control of bladder voiding (Fowler et al., 2008; de Groat and Yoshimura, 2009). Whilst the urothelium has been shown to exhibit sensory functions (Birder and Andersson, 2013), the primary role of the urothelium is to provide a blood/urine barrier between the potentially toxic levels of urea, ammonia, and other noxious metabolites found within the urine and the underlying interstitium of the bladder (Lasič et al., 2015).

The considerable transcellular and paracellular impermeability of the urothelial barrier is maintained by tight junctions between luminally adjacent urothelial cells, hydrophobic uroplakin plaques, and a substantial glycosaminoglycan (GAG) mucus layer, that block the movement of ions, solutes and toxic metabolites (Hurst et al., 2015; Lasič et al., 2015; Grundy et al., 2018a). In a healthy bladder, these components form the least penetrable epithelium found in mammals (Lasič et al., 2015), however, a number of studies have shown patients with IC/BPS have a diminished urothelium or urothelial barrier (Parsons, 2007; Hurst et al., 2015). It has therefore been hypothesised that in cases of increased urothelial permeability, the myriad of noxious molecules contained within the urine have direct access to the underlying sensory afferents to induce neuronal hyperexcitability and induce exaggerated sensation and bladder dysfunction (Parsons, 2007; Gonzalez et al., 2014; Yoshimura et al., 2014). To further protect the underlying structures of the bladder from increases in urothelial permeability the urothelium consists of three cell layers. These include a basal layer of cells located furthest from the bladder lumen that includes stem cells responsible for the generation and proliferation of new urothelium (Shin et al., 2011; Wang et al., 2017). Partially differentiated intermediate cells are located between the apical umbrella cells and the basal cells, providing a reservoir of readily available replacement cells in the event of umbrella cell damage or infection that provokes sloughing (Shin et al., 2011; Hurst et al., 2015; Wang et al., 2017) and enables rapid restoration of bladder impermeabiilty following damage. *In vivo* bladder instillation of protamine sulfate is a commonly used method for experimentally inducing bladder permeability in the absence of overt bladder inflammation or significant urothelial damage that returns to normal over a period of 3-7 days (Lavelle et al., 2002; Shin et al., 2011; Greenwood-Van Meerveld et al., 2015).

It remains to be determined whether bladder permeability is part of the initiating pathophysiology of bladder hypersensitivity in IC/PBS patients or a downstream consequence of inflammation that further exacerbates the condition. In this study we sought to determine if chronic sensitisation of bladder afferents could be induced by specifically increasing urothelial permeability by using an *in vivo* intrabladder infusion of protamine sulfate.

## Materials and Methods

### Animals

12- to 16- week-old female C57BL/6J mice were used in this study. The South Australian Health and Medical Research Institute (SAHMRI) ethics committee approved all experiments involving animals (animal ethics number SAM234). Mice were acquired from an in-house C57BL/6J breeding program (JAX strain #000664; originally purchased from The Jackson Laboratory; breeding barn MP14; Bar Harbor, ME) within SAHMRI’s specific and opportunistic pathogen-free animal care facility. Prior to bladder infusion with either protamine sulfate or vehicle (saline), mice were group housed (5 mice per cage) in individual ventilated cages filled with chip coarse dust-free aspen bedding (Cat#–ASPJMAEBCA; PuraBed, Niederglatt, Switzerland). Mice had free access to LabDiet JL Rat and Mouse/Auto6F chow (Cat#5K52, Jackson Laboratories, St. Louis, MO) and autoclaved reverse osmosis water within a temperature-controlled environment of 22°C and a 12 hour light/12 hour dark cycle. Following *in vivo* procedures, mice were individually housed (1 mouse per cage) in individual ventilated cages. Mice had an average weight of 21–23 g on the day of experimentation. *Ex vivo* afferent recordings and myeloperoxidase (MPO) analysis were performed on separate mice at 16 weeks of age.

Five groups of mice were used in this study. (**1**) mice that received no treatment (Control: N = 5). (**2**) Mice that received intrabladder infusion of saline and used 1 day later (1-day post vehicle: N = 6). (**3**) Mice that received intrabladder infusion of protamine sulfate and used 1 day later (1-day post PS: N = 5). (**4**) Mice that received intrabladder infusion of protamine sulfate and used 7 days later (7 days post PS: N = 5). (**5**) Mice that received intrabladder infusion of protamine sulfate and used 28 days later (28 days post PS: N = 6).

### *In vivo* Infusion of Protamine Sulfate

The protamine sulfate model used was ostensibly as described previously (Greenwood-Van Meerveld et al., 2015; Mohammadi et al., 2018). These studies have shown that bladders from protamine sulfate-treated animals showed signs of oedema at 1–5 days post infusion, but the urothelium remains intact with no damage to the umbrella cells. Also, at 1 and 3 days post-infusion, there are significant decreases in transepithelial electrical resistance in protamine sulfate-treated urinary bladders which normalises 5 days post-infusion (Greenwood-Van Meerveld et al., 2015). In the current study, mice (12–16 weeks) were anaesthetised (isoflurane 2%-4% in oxygen) and a lubricated catheter (PE 50 tubing) was inserted into the urethra. Urine was removed using a suction syringe, and a new lubricated catheter (PE 50 tubing) was inserted. A fine silk suture was used to tie around the catheter and the urethral opening to secure the tube and to limit leakage of solution through the urethra during infusion. The bladder was then infused with either 100 μl of protamine sulfate (1 mg/ml) (Sigma-Aldrich) dissolved in isotonic saline, or 100 μl of isotonic saline (vehicle) via a syringe. Mice were maintained under anaesthetic for 10 minutes with either the protamine sulfate or vehicle held within the bladder. After 10 minutes, the bladder was drained via the syringe, the syringe was removed and the mice were allowed to recover, housed individually, and monitored twice daily for two days or until mice were humanely killed (1, 7, or 28 days post procedure) for use in bladder afferent recordings or in the myeloperoxidase assay (Grundy et al., 2018c).

### *Ex Vivo* Bladder Afferent Recordings

Bladder afferent nerve recordings were performed using an *ex vivo* model previously characterised (Grundy et al., 2018b–d; Grundy et al., 2020) on 16 week old mice that either did or did-not (control mice) receive intrabladder infusion of protamine sulfate or vehicle. Mice were humanely killed with CO_2_ and dissected under a microscope with continual perfusion of gassed (95% O2, 5% CO2) Krebs-bicarbonate solution (composition in mmol/L: NaCl 118.4, NaHCO_3_ 24.9, CaCl_2_ 1.9, MgSO_4_ 1.2, KCl 4.7, KH2PO_4_ 1.2, glucose 11.7) at 35°C. A catheter attached to a syringe pump was inserted into the bladder via the urethra to allow controlled distension at 100 μl/min with saline (0.9%). A second two-way catheter was inserted into the bladder dome, which is attached to a pressure transducer and an outflow tap, enabling the recording of intravesical pressure during bladder distension and the ability to passively drain the bladder after each distension. Pelvic afferents, isolated from nerve fibres between the pelvic ganglia and the spinal cord, were dissected into fine multiunit branches and inserted into a glass electrode. Multiunit afferent activity was detected by a Neurolog headstage (NL100, Digitimer Ltd, United Kingdom), amplified, filtered (NL125, band pass 300–4000 Hz) and captured by a computer via a power 1401 interface and Spike 2 software (version 5.2.1, CED, United Kingdom).

### Experimental Protocol

A single nerve bundle was isolated for each experiment (N = 1 per mouse) and inserted into the glass electrode during the dissection process. This nerve bundle was used for the entire experiment allowing multiunit afferent nerve recordings to be performed. Bladder distensions (100 μl/min) were performed from 0 to 50 mmHg at 10-minute intervals until the afferent response to distension was stable and reproducible.

### Experimental Analysis

The raw nerve activity in *ex vivo* afferent recordings was determined by the number of action potentials passing a pre-set threshold (twice the background electrical level). This ensures only mechanosensitive afferents are recorded. Each afferent nerve contains a different number of single afferent units. In order to identify individual units, single unit analysis was performed offline by matching individual spike waveforms through linear interpolation using Spike 2 software. Baseline afferent sensitivity was determined by averaging the baseline nerve activity occurring between distensions over a 5 minute period. Single afferent units were characterised as previously described (Grundy et al., 2019b). Briefly, afferent units were deemed ‘low threshold’ if the pressure required to elicit continuous action potential firing was 16 mmHg or less. In contrast, ‘high threshold’ afferents exhibited continuous action potential firing only when pressures exceed 18 mmHg (Grundy et al., 2019b; Grundy et al., 2020).

### Myeloperoxidase (MPO) Assay

Bladders isolated from mice immediately following humane euthanasia with CO_2_ were sectioned longitudinally, rinsed in ice-cold PBS, and snap frozen in liquid nitrogen (Grundy et al., 2018c). Tissue was defrosted on ice, weighed, and put in 1.5 ml HTAB buffer (0.5% Hexadecyltrimethylammonium bromide, pH6): 2.5 g HTAB (H5882, Sigma, Australia) added to 500 ml of 50 mM PBS, pH6). The tissue was then homogenised with a cooled TissueLyser^TM^ (Qiagen, Hilden, Germany) and sonicated for 1 min. 20 μl of tissue homogenate was added to 200 μl of an o-dianisidine dihydrochloride (o-dianisidine) solution containing H_2_O_2_. Absorption data was recorded on a VERSAmax 20 plate reader (Molecular Devices) at 450 nm wavelength for 4 min with 30 sec interval-reading. All samples were run in triplicates and a blank was included.

### Analysis and Statistics

Data are expressed as Mean ± SEM. N equals the number of animals, whilst n equals the number of individual afferents identified using single unit analysis. Differences were considered significant at a level of ^∗^*P*< 0.05, ^∗∗^*P* < 0.01,^∗∗∗^*P* < 0.001. All data were analysed using GraphPad Prism 7 (San Diego, CA, USA) in a blinded manner. For bladder afferent responses to distension, compliance curves and multiunit and single unit data was found to be normally distributed and analysed using a two-way ANOVA with Tukey’s multiple comparisons. The number of single units per experiment, activation thresholds, peak afferent firing and MPO concentrations were analysed by one-way ANOVA with Tukey’s multiple comparisons. The specific tests used to analyse each data set and N numbers are indicated within the individual figure legends.

## Results

**FIGURE 1:**
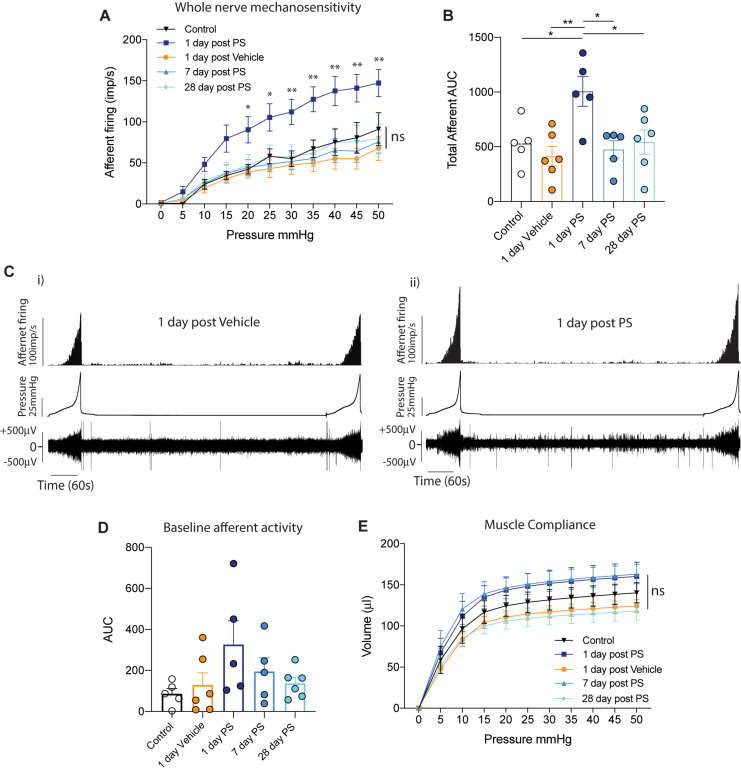
*In vivo* protamine sulfate treatment sensitises bladder afferent responses to distension. **(A)** One day after *in vivo* infusion of protamine sulfate (PS), but not vehicle, bladder afferents exhibit significant hypersensitivity to bladder distension (N = 5–6 mice, *P < 0.05, **P < 0.01, Two-way ANOVA with Tukey’s multiple comparisons *post hoc* test). Bladder afferent mechanosensitivity is normalised at seven days post PS infusion and is maintained at control levels 28 days after PS infusion (N = 5–6 mice, ^*ns*^ *P* > 0.05, Two-way ANOVA with Tukey’s multiple comparisons *post hoc* test). **(B)** Total afferent area under the curve (AUC) is a summation of peak afferent firing from each 5 mmHg pressure increment from 0–50 mmHg. Total afferent AUC is significantly elevated one day after *in vivo* infusion of protamine sulfate (PS), but not after vehicle treatment (N = 5–6 mice, *P < 0.05, **P < 0.01, One-way ANOVA with Tukey’s multiple comparisons *post hoc* test). **(C)** Example experimental traces showing bladder afferent firing, intravesical pressure, and raw nerve activity to repeated distensions with saline (i) 1 day after bladder infusion with vehicle and (ii) 1 day after bladder infusion with protamine sulfate. Afferent responses to distension (0–50 mmHg) are stable and reproducible. **(D)** Spontaneous baseline afferent responses (N = 5–6 mice, ^*ns*^ *P* > 0.05, One-way ANOVA with Tukey’s multiple comparisons *post hoc* test). **(E)** The compliance (pressure/volume relationship) of the bladder muscle during graded bladder distension is not significantly altered following intra-bladder infusion of PS or vehicle (N = 5–6 mice, ^*ns*^*P* > 0.05, Two-way ANOVA with Tukey’s multiple comparisons *post hoc* test).

As increases in the permeability or breakdown of the bladder urothelium have commonly been associated with IC/BPS symptoms, and it is known that bladder afferent sensory neurons are located in close proximity to the urothelium (Spencer et al., 2018), we hypothesised that the bladder hyperalgesia occurring with increased urothelial permeability is via a sensitisation of these afferent neurons to bladder distension. To test this hypothesis, we performed *ex vivo* recordings from bladder afferents (Figure 1). We compared bladder afferent mechanosensitivity from mice 1 day after *in vivo* instillation of vehicle with mice 1, 7, and 28 days after *in vivo* instillation of protamine sulfate. We observed a significant increase in bladder afferent mechanosensitivity from bladders 1 day after protamine sulfate treatment, but no change in mechanosensitivity from mice receiving the sham vehicle infusion (Figures 1A-C). Only low levels of spontaneous bladder afferent firing were observed in the periods between bladder distension (Figure 1C) and this was not found to be significantly altered following vehicle or protamine sulfate treatment (Figure 1D). The increase in bladder afferent mechanosensitivity we observed at 1-day post protamine sulfate treatment occurred in the absence of changes in muscle compliance (pressure/volume relationship; Figure 1E), indicating that the effects of protamine sulfate on afferent mechanosensitivity are not secondary to changes in muscle function. Intriguingly, the bladder afferent hypersensitivity to graded distension that was observed 1-day post protamine sulfate returned to control levels at day 7 post treatment and was maintained at control levels up to 28 days post treatment (Figures 1A–E). These data therefore indicate that protamine sulfate induced urothelial permeability induces only transient afferent hypersensitivity.

**FIGURE 2:**
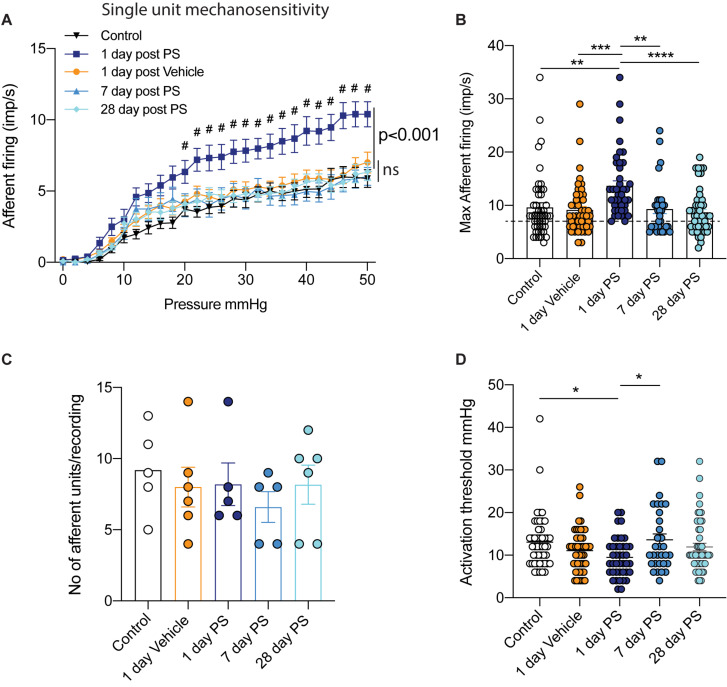
Single unit bladder afferent mechanosensitivity. **(A)** One day after *in vivo* infusion of protamine sulfate (PS), but not vehicle, single bladder afferent units exhibit hypersensitivity to bladder distension (n = 41–48 individual units from N = 5–6 mice, ^#^*P* < 0.05, Two-way ANOVA with Tukey’s multiple comparisons *post hoc* test). Single unit bladder afferent mechanosensitivity is unchanged at seven days post PS infusion and is maintained at control levels 28 days after PS infusion (n = 31–49 individual units N = 5–6 mice, ^*ns*^ *P* > 0.05, Two-way ANOVA with Tukey’s multiple comparisons *post hoc* test). **(B)** Peak single unit afferent responses to bladder distension (0–50 mmHg) are significantly elevated one day after PS infusion compared to control, One day post vehicle, and seven and 28 days post PS (n = 31–49 individual units from N = 5–6 mice, **P < 0.01,***P < 0.05, one-way ANOVA with Tukey’s multiple comparisons *post hoc* test). Peak firing of each individual unit is * represented by a single dot. Dashed line represents least responsive units in 1-day PS group. **(C)** The mean number of single units differentiated by spike sorting software in each individual multiunit nerve recording experiment (N = 5–6 mice). **(D)** The activation threshold of individual afferent units is significantly reduced one day after *in vivo* PS but not vehicle infusion (n = 31–49 individual units from N = 5–6 mice, *P < 0.05, one-way ANOVA with Tukey’s multiple comparisons *post hoc* test).

*Post hoc* single-unit analysis of multiunit bladder afferent recordings allows for a more comprehensive understanding of the entire mechanosensitive response. Upon grouping all single afferent units from each experimental cohort together, we found the same pattern of response as seen in our multiunit afferent responses (Figure 1), with a significant increase in mechanosensitivity during the course of graded bladder distension (Figure 2A). Additionally, when we analysed the peak afferent response to bladder distension (0-50 mmHg) from each individual afferent unit we discovered that afferents recorded at 1-day post protamine sulfate exhibited a significant increase in the peak firing frequency (Figure 2B). Furthermore, analysing this data further shows us that whilst the lowest peak afferent firing in mice 1-day post protamine sulfate is 7 imp/s, in the other experimental cohorts, a large proportion of units exhibit peak firing frequencies between 2-7 imp/s (Figure 2B). To identify if the transient increases in afferent firing following protamine sulfate treatment were due to the recruitment of an additional cohort of afferents (silent afferents), as has been identified in the presence of inflammatory mediators (Michaelis et al., 1996; Prato et al., 2017; Grundy et al., 2020), we compared the number of individual single units identified by spike waveform analysis per experiment. We found no significant difference in the number of single units per experiments between experimental cohorts (Figure 2C). In addition to an increase in mechanosensitive bladder afferent firing frequency 1-day after protamine sulfate treatment, we also found single units exhibit significantly reduced activation thresholds to bladder distension compared to control and 7-days after protamine sulfate (Figure 2D), thus firing the first train of action potentials at lower distension pressures.

**FIGURE 3:**
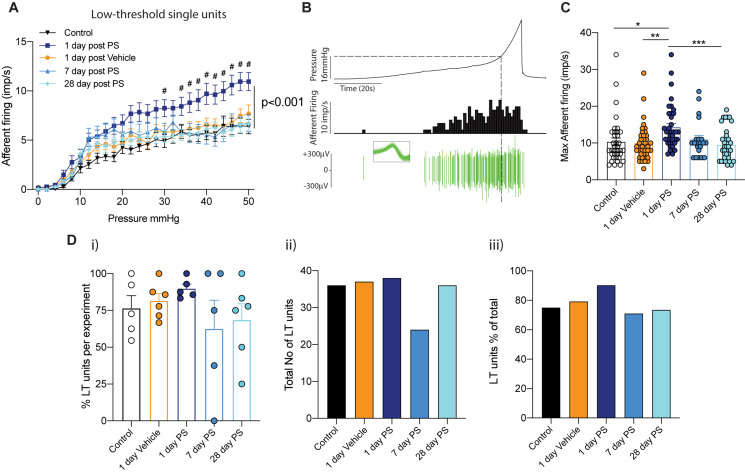
Low-threshold bladder afferent responses following *in vivo* protamine sulfate treatment. **(A)** Low-threshold bladder afferent mechanoreceptors exhibit significant hypersensitivity to bladder distension one day after *in vivo* infusion of protamine sulfate (PS) but not vehicle (n = 21–38 individual units from N = 5–6 mice, ^#^*P* < 0.05, Two-way ANOVA with Tukey’s multiple comparisons *post hoc* test). **(B)** Example experimental trace showing a single low-threshold afferent unit in response to graded bladder distensions with saline (0–50 mmHg). Dashed line represents distinction of activation thresholds between low- and high- threshold units (16 mmHg). Individual units are determined by color spike profile (insert). **(C)** Peak afferent response of low-threshold bladder afferents to distension (*P < 0.05, **P < 0.01, **P < 0.001, One-way ANOVA with Tukey’s multiple comparisons *post hoc* test). **(D)** (i) The percentage of single afferent units classified as low-threshold (LT) per experiment, (ii) the total number of units classified as low-threshold (LT), and (iii), low-threshold units (LT) as a percentage of the total number of mechanosensitive units in each experimental cohort.

**FIGURE 4:**
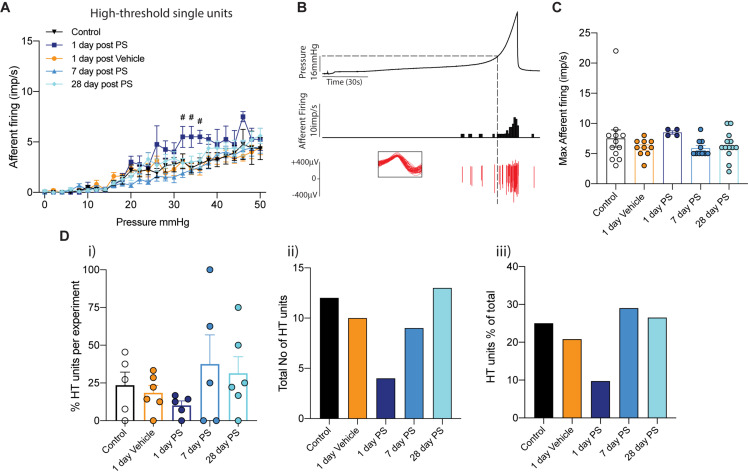
High-threshold bladder afferent responses following *in vivo* protamine sulfate treatment. **(A)** High-threshold bladder afferent mechanoreceptors exhibit moderate hypersensitivity to bladder distension one day after *in vivo* infusion of protamine sulfate but not vehicle (PS) (n = 4–13 individual units from N = 5–6 mice, ^#^*P* < 0.05, Two-way ANOVA with Tukey’s multiple comparisons *post hoc* test). **(B)** Example experimental trace showing a single high-threshold afferent unit in response to graded bladder distensions with saline (0–50 mmHg). Dashed line represents distinction of activation thresholds between low- and high- threshold units. Activation threshold is determined as the pressure from which a continuous train of action potentials occurs. Individual units are determined by color spike profile (insert). **(C)** Peak afferent response of high-threshold bladder afferents to distension (ns, *P* > 0.05. One-way ANOVA with Tukey’s multiple comparisons *post hoc* test). **(D)** (i) The percentage of single afferent units classified as high-threshold (HT) per experiment, (ii) the total number of units classified as high-threshold (HT), and (iii), high-threshold units (HT) as a percentage of the total number of mechanosensitive units in each experimental cohort.

In both our multiunit afferent recordings and following *post hoc* single unit analysis, we observed an overall sensitisation of mechanosensitive afferents 1-day after protamine sulfate infusion, but not vehicle, that was most obvious above 20 mmHg bladder distension (Figures 1, 2). In order to investigate this further, we distinguished functional subpopulations of low- and high-threshold mechanosensitive units by activation thresholds as previously described (Grundy et al., 2019b) (Figures 3, 4). When we dissect apart these single units, it becomes clear that not all units are equally sensitive to changes in bladder permeability. At 1-day post-protamine sulfate, low-threshold units exhibited hypersensitivity to graded bladder distension (Figure 3A) and an increase in the peak afferent response to distension (Figures 3B,C). Overall, there were no significant changes in the number of low-threshold afferents per experiment (Figures 3D i–iii). In contrast to low-threshold afferents, the mechanosensitivity of high-threshold afferents was relatively unaffected by protamine sulfate (Figure 4A) and did not increase the peak afferent response to distension (Figures 4B,C). There were also no significant changes in the number of high-threshold afferents, but there was a trend toward a decrease in the total number and proportion of afferent units that could be classified as high threshold at 1-day post-protamine sulfate (Figures 4D i–iii).

**FIGURE 5:**
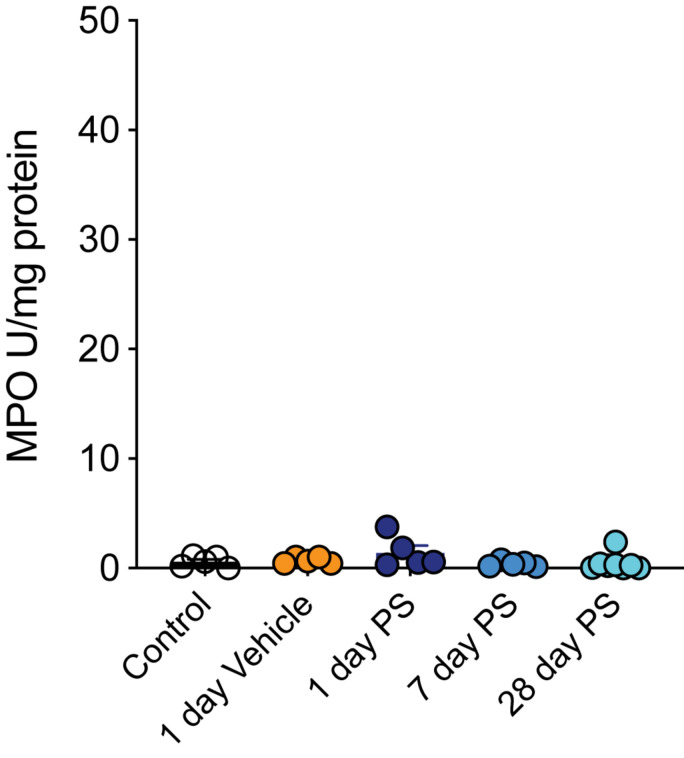
Protamine sulfate does not induce overt bladder inflammation. *In vivo* bladder infusion with protamine sulfate or vehicle has no effect on bladder myeloperoxidase (MPO) concentrations at 1, 7, or 28 days post infusion (N = 5–8 mice, ^*ns*^ *P* > 0.05, One-way ANOVA with Tukey’s multiple comparisons *post hoc* test).

To confirm that the changes in bladder afferent mechanosensitivity we observed in response to protamine sulfate infusion were not due to the induction of inflammation, a known inducer of neuronal hypersensitivity (de Groat and Yoshimura, 2009), we measured myeloperoxidase (MPO) activity in bladders isolated from each cohort of mice (Figure 5). Overall, we found that neither the sham procedure (vehicle) nor protamine sulfate infusion had a significant effect on bladder MPO, indicating an absence of overt inflammation at the time points tested.

## Discussion

Bladder afferent input into the central nervous system is required for normal bladder function, regulating the autonomic circuits that prompt detrusor relaxation during storage, and initiating urge sensations necessary for the conscious control of voiding (Fowler et al., 2008). As the degree of bladder afferent input into the central nervous system is correlated both to the intensity of sensation and autonomic output controlling the micturition reflex, an increase in bladder permeability and the corresponding increase in afferent firing has the potential to translate to the sensory symptoms of urgency, frequency and pelvic pain associated with IC/BPS (Fowler et al., 2008; de Groat and Yoshimura, 2009; Yoshimura et al., 2014). Accordingly, preclinical models of bladder inflammation and infection that induce changes in the intensity of bladder afferent signalling have been correlated with bladder dysfunction (de Groat and Yoshimura, 2009; Grundy et al., 2018a).

There is considerable evidence that the pathophysiology of IC/BPS incorporates an increase in urothelial permeability (Hurst et al., 2015). Numerous studies, both clinical and pre-clinical, have provided evidence of urothelial abnormalities in IC/BPS (Parsons, 2007; Hurst et al., 2015). However, delineating bladder permeability from bladder inflammation, as well as cause and effect, has proved challenging (Grundy et al., 2019a). Inflammation is a well-known regulator of neuronal excitability (Grundy et al., 2018a), and our aim was to investigate only the role of increasing bladder permeability in bladder afferent sensitisation. To achieve this, we used a brief intrabladder infusion of 1 mg/ml protamine sulfate to induce bladder permeability. Whilst we didn’t specifically demonstrate this in the current study, this concentration has previously been shown to induce bladder permeability without overt bladder inflammation or urothelial damage (Lavelle et al., 2002; Shin et al., 2011; Greenwood-Van Meerveld et al., 2015). Protamine sulfate promotes an increase in bladder permeability by inactivating the sulphated polysaccharides of the GAG layer, increasing transcellular permeability of the urothelium and thus increasing absorption of urine solutes (Lasič et al., 2015). Crucially, in this study we confirmed that neither protamine sulfate nor vehicle treatment induced bladder inflammation by quantifying myeloperoxidase (MPO), a key local mediator of tissue damage and inflammation (Aratani, 2018) used to quantify bladder inflammation in both pre-clinical and clinical samples (Oottamasathien et al., 2011; Ciragil et al., 2014). Overall, we show that *in vivo* bladder infusion of protamine sulfate significantly enhances bladder afferent mechanosensitivity to graded distension in the absence of inflammation. These data provide the first direct evidence for a role of bladder permeability in regulating bladder mechanosensation. However, we identify that this neuronal hyperexcitability is transient, with bladder afferent responses returning to control levels within 7 days after protamine sulfate treatment. We also show that bladder afferent responses are maintained at control levels 28-days post-protamine sulfate, suggesting secondary hypersensitivity is not occurring in these conditions.

A number of previous studies have provided indirect evidence that changes in bladder permeability can induce sensory dysfunction (Keay et al., 2014; Hurst et al., 2015; Grundy et al., 2018a; Mohammadi et al., 2018), however, it remained to be determined if bladder permeability is part of the underlying pathology of visceral hypersensitivity disorders such as IC/BPS, or an unavoidable consequence of inflammation (Grundy et al., 2019a). Our data show that increasing bladder permeability with protamine sulfate in the absence of inflammation induces hypersensitivity of bladder afferents to graded bladder distension. It is likely these changes in bladder afferent sensitivity are responsible for the pelvic sensitivity observed in response to suprapubic stimulation following protamine sulfate treatment (Mohammadi et al., 2018). We also observed increased peak firing of single units 1-day after protamine sulfate treatment, as well as a trend toward decreased afferent activation thresholds and an increase in the ratio of low-threshold afferents. We did not observe an increase in the number of mechanosensory single units per experiment in any of the experimental groups, indicating that ‘silent afferents’ were not recruited by protamine sulfate treatment. Silent afferents represent a functionally distinct subclass of bladder afferents that have traditionally been recruited to become mechanosensitive in the presence of inflammatory stimuli (Habler et al., 1990; Dmitrieva and McMahon, 1996; Grundy et al., 2020; Guo et al., 2020). The lack of silent afferent recruitment here further supports our hypothesis that protamine sulfate-induced hypersensitivity is functionally distinct from inflammatory-induced hypersensitivity. Together these data indicate that increasing bladder permeability is able to potentiate the mechanosensory signal conveyed to the spinal cord during bladder distension and that this effect at this concentration of protamine sulfate (1 mg/ml) is transient. Whilst we did not investigate the precise mechanism driving bladder afferent sensitivity in this study, it is likely that as urothelial permeability is increased, urinary contents gain access to afferent nerves to either cause afferent hypersensitivity directly, or indirectly via actions on urothelial cells. We and others have shown that a variety of endogenous mediators can activate bladder afferents, causing afferent hypersensitivity (de Groat and Yoshimura, 2009; Birder and Andersson, 2013; Yoshimura et al., 2014; Grundy et al., 2018b; Konthapakdee et al., 2019; Grundy et al., 2020).

Interestingly, a previous study using 5x the concentration of protamine sulfate demonstrated that visceromotor responses (VMR) to bladder distension were actually reduced at 1-day post infusion (Stemler et al., 2013). This particular study showed the reduction in VMR was associated with a decrease in bladder expression of the pro-inflammatory/pro-nociceptive cytokine IL-1ß and an increased expression of the anti-inflammatory cytokine IL-4. The authors also suggest that the bladder injury induced by the higher concentration of protamine sulfate could increase the renewal mechanisms originating from transient amplifying regulation rather than urothelial stem cell activation (Stemler et al., 2013). As afferent sensitisation is a balance between pro- and anti-nociceptive mechanisms (Brierley and Linden, 2014; Sadeghi et al., 2018; Grundy et al., 2019a), the Stemler study, at 5x the concentration of protamine sulfate, may have recruited more anti-nociceptive mechanisms than we have seen in our current study.

Interestingly in our study, and in contrast to the proposed hypothesis that changes in bladder permeability are an initiating factor in the development of IC/BPS (Parsons, 2007), the bladder afferent response to distension returned to control levels by day 7 after protamine sulfate treatment. As inflammation was not present within the bladder, we hypothesise that this recovery was due to rapid repair of the urothelial barrier. The urothelium has the tightest epithelial barrier of all mammalian epithelial barriers (Lasič et al., 2015), necessary for the maintenance of the blood-urine barrier and compartmentalisation of the toxic constituents of urine within the bladder during storage. Under normal conditions, urothelial cells have an extremely slow turnover rate (Birder and de Groat, 2007), however, following injury or infection, the urothelium undergoes rapid injury-induced proliferation of urothelial stem cells to restore the tight epithelial barrier (Shin et al., 2011; Wang et al., 2017). Indeed, studies using either the same or higher concentrations of protamine sulfate as in our experiments have shown that the barrier function of the urothelium is restored within 2-5 days after injury (Lavelle et al., 2002; Greenwood-Van Meerveld et al., 2015). These data thus indicate that transient increases in bladder permeability, such as in this study, are unable to induce a protracted state of bladder permeability or bladder afferent hypersensitivity. Given that IC/BPS is chronic in nature, our data suggests that additional pathophysiology, or repeated insults to the bladder, are required to induce long term neuroplasticity and the induction of chronic afferent hypersensitivity necessary for sensory and urological dysfunction (de Groat and Yoshimura, 2009; Grundy et al., 2018a). Indeed, a positive feedback mechanism has been proposed to exist, incorporating urothelial barrier disruption, inflammation, and neuronal hyperexcitability in the induction of IC/BPS (Gonzalez et al., 2014; Yoshimura et al., 2014). A role for inflammation in mediating bladder afferent hyperexcitability is well established (Yoshimura et al., 2014; Montalbetti et al., 2017; Grundy et al., 2018a; Grundy et al., 2019a), and a number of pre-clinical studies have shown that inflammation is able to induce urothelial permeability (Lavelle et al., 1998; Eichel et al., 2001; Grundy et al., 2018a; Grundy et al., 2019a). Therefore, our data supports a mechanism whereby chronic bladder permeability is likely a downstream consequence of inflammation, that provides an addition degree of neuronal sensitisation.

## Conclusion

Our data indicate that whilst changes in bladder permeability are able to induce transient bladder afferent hypersensitivity, highly regulated homeostatic mechanisms exist in a murine model to rapidly repair the urothelial barrier, and as such, it is unlikely that changes in permeability alone are the initiating factor in the development of bladder disorders such as IC/BPS.

## Data Availability Statement

The raw data supporting the conclusions of this article will be made available by the authors, without undue reservation.

## Ethics Statement

The animal study was reviewed and approved by South Australian Health and Medical Research Animal Ethics Committee Project approval number: SAM234.

## Author Contributions

LG, SMB, GH, and BG, conceived and designed research. EM provided essential technical assistance in bladder infusions. LG and AC performed *in vivo* bladder infusions. LG conducted *ex vivo* afferent recordings, analysis, made figures, and wrote the manuscript. AL performed MPO analysis. All authors provided feedback and revision of the final manuscript.

## Conflict of Interest

The authors declare that the research was conducted in the absence of any commercial or financial relationships that could be construed as a potential conflict of interest.
